# Quantification of thickness and wrinkling of exfoliated two-dimensional zeolite nanosheets

**DOI:** 10.1038/ncomms8128

**Published:** 2015-05-11

**Authors:** Prashant Kumar, Kumar Varoon Agrawal, Michael Tsapatsis, K. Andre Mkhoyan

**Affiliations:** 1 Department of Chemical Engineering and Materials Science, University of Minnesota, Minneapolis, Minnesota 55455, USA

## Abstract

Some two-dimensional (2D) exfoliated zeolites are single- or near single-unit cell thick silicates that can function as molecular sieves. Although they have already found uses as catalysts, adsorbents and membranes precise determination of their thickness and wrinkling is critical as these properties influence their functionality. Here we demonstrate a method to accurately determine the thickness and wrinkles of a 2D zeolite nanosheet by comprehensive 3D mapping of its reciprocal lattice. Since the intensity modulation of a diffraction spot on tilting is a fingerprint of the thickness, and changes in the spot shape are a measure of wrinkling, this mapping is achieved using a large-angle tilt-series of electron diffraction patterns. Application of the method to a 2D zeolite with MFI structure reveals that the exfoliated MFI nanosheet is 1.5 unit cells (3.0 nm) thick and wrinkled anisotropically with up to 0.8 nm average surface roughness.

## Abstract (web summary)

Two-dimensional sheets of zeolites can function as molecular sieves for applications such as membranes or as catalysts. Here, the authors demonstrate a method using electron diffraction patterns to accurately measure the thickness and wrinkles of thin zeolite nanosheets.

## Graphical abstract

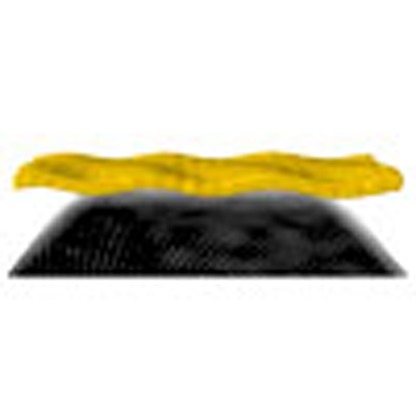

Zeolites are three-dimensional (3D) framework silicates with precisely sized pores of molecular dimensions^1^. Two-dimensional (2D) zeolites and zeolite nanosheets^2^,^3^,^4^ with single, double or fractional unit cell (UC) thickness are particularly desirable for separation^5^,^6^ and catalysis of bulky molecules^7^,^8^,^9^,^10^. They may also emerge as candidates for device fabrication requiring low-*k* dielectric materials^11^.

Since their structure is crucial in order to predict or interpret their adsorption, transport and catalytic properties^10^,^12^,^13^, and can vary depending on the synthesis procedure^14^,^15^, its quantification is highly desirable. Unlike many 2D materials, such as graphene, BN, phosphorene, MoS_2_ and other transition metal dichalcogenides, where the UC is 1-, 2- or 3-atom-layers thick, the UC of zeolites can be >10-atom-layers thick. Moreover, zeolite nanosheets can be synthesized with thicknesses that are non-integer multiples of the UC thickness^5^. Therefore, methods developed to characterize other 2D materials are not necessarily applicable to 2D zeolites.

Thickness measurements of 2D materials are often performed by atomic force microscopy (AFM)^5^,^16^,^17^, X-ray reflectivity experiments^18^,^19^,^20^ or imaging cross-sectional samples with conventional transmission electron microscopy (TEM)^8^. However, AFM data cannot provide crystallographic information over the entire nanosheet thickness, and X-ray reflectivity measurements often require fabrication of a periodic multilayer, which is not feasible for nanosheets with sub-micron lateral dimensions such as 2D zeolites. Preparation of cross-sections for TEM imaging can be challenging, and conventional TEM images require in-depth analysis as contrast is strongly sensitive to imaging conditions and specimen thickness^21^. Moreover, zeolites are electron beam-sensitive materials that suffer from knock-on damage and radiolysis at high and low accelerating voltages^22^. Therefore, a method for unambiguously determining the thickness and structure of 2D zeolites remains elusive.

In addition to thickness, it is also important to determine deviations from the nominal (ideal, wrinkle-free) structure of 2D zeolites. These deviations can affect their internal and external surface structure and pore openings, which, in turn, can also affect their adsorption, transport and catalytic properties. Atomic-scale wrinkles have been observed in 2D materials such as graphene^23^ through lattice imaging using scanning TEM (STEM)^24^,^25^,^26^,^27^. However, using this approach for zeolites is challenging due to their rapid amorphization under the required high-dose electron irradiation.

Here we demonstrate a method that, based on a set of TEM experiments, provides complete quantitative characterization of the atomic structure of zeolite nanosheets, that is, crystal structure and uniformity through high-resolution TEM (HR-TEM) and annular dark-field (ADF)-STEM imaging, and thickness and wrinkling through electron diffraction. Although applicable for all 2D materials, it is particularly well suited for 2D zeolites. The all-silica 2D zeolite with the MFI structure type (for a list of structure types see http://www.iza-online.org) is used as the prototype material^5^,^8^ and its anisotropic wrinkling is quantified.

## Results

### In-plane structure determination

**Figure 1:**
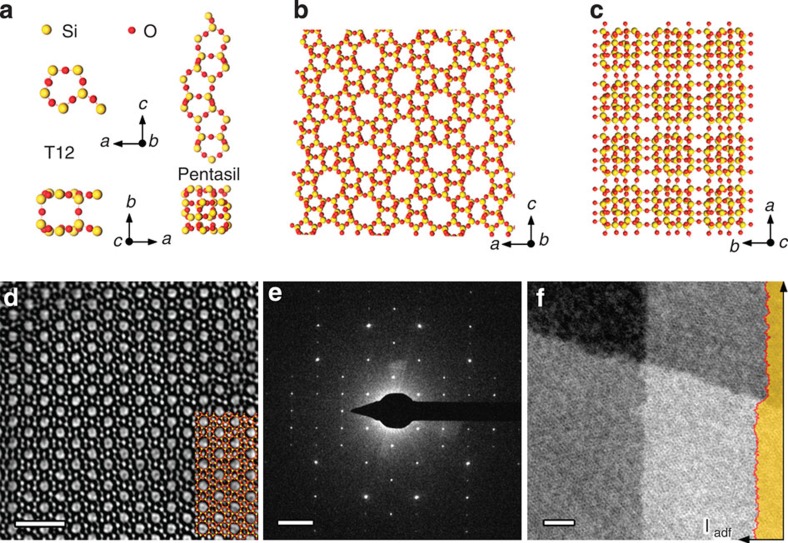
MFI nanosheet crystal structure. (**a**) Construction of MFI pentasil chain from the smallest T12 building unit, which contains 12 silicon atoms. (**b**) Projection of MFI crystal structure along *b*-direction formed by linking pentasil chains. MFI nanosheets are typically ∼100–200 nm wide along *a*- and *c*-directions. (**c**) Projection of MFI nanosheet along *c*-direction showing three complete pentasil chains in *b*-direction (1.5 UCs along *b*-direction). (**d**) Bragg-filtered HR-TEM image of MFI nanosheet with the overlaid crystal structure along [010] direction. (**e**) Diffraction pattern of MFI nanosheet along [010] zone axis. (**f**) HAADF-STEM image of two overlaid MFI nanosheets with intensity-scan taken from the overlaid area. It shows homogenous thickness across nanosheets and doubling of intensity (that is, doubling of thickness) in the overlaid region. Scale bars, 3 nm (**d**), 1 nm^−1^ (**e**) and 10 nm (**f**).

MFI belongs to the pentasil family of zeolites, where the periodic building unit is composed of 12 interconnected SiO_4_ tetrahedral (T12) units^28^ (Fig. 1a). Rotation of T12 units about the *c* axis (right- or left rotation), along with translation by half of a UC in the *c*-direction, forms right-handed or left-handed pentasil chains consisting of five membered rings. Alternating left- and right-handed chains, when connected along the *a*-direction through inversion symmetry, form the MFI zeolite^29^ structure with an orthorhombic UC (Fig. 1b,c and Supplementary Fig. 1).

The crystal structure of MFI nanosheets was assessed by a combination of HR-TEM imaging, electron diffraction pattern analysis and high-angle annular dark-field STEM (HAADF-STEM) imaging. The HR-TEM image viewed along the [010] crystallographic direction (Fig. 1d) and the [010] zone axis electron diffraction pattern (Fig. 1e) are in agreement with the standard bulk MFI structure, confirming preservation of bulk crystal structure in nanosheets. Thickness-sensitive HAADF-STEM images (Fig. 1f) reveal that the synthesized nanosheets used in this study are uniform and have the same thickness. As can be seen from Fig. 1f, the intensity of ADF image doubles between the areas containing single nanosheets to that with two overlapped nanosheets. While these experiments provide unambiguous determination of crystal structure, they are not useful for thickness evaluation.

### Quantification of thickness

**Figure 2:**
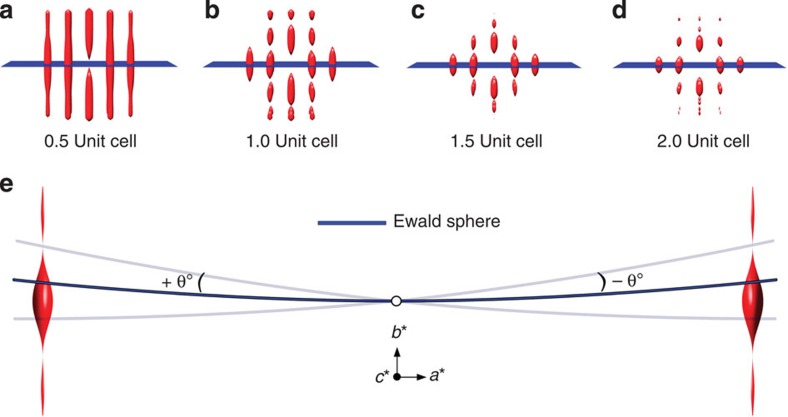
Thickness dependence of rel-rods. Isosurfaces of reciprocal lattices at 5% of maximum intensity simulated for four crystal models of MFI nanosheets with thickness of (**a**) 0.5, (**b**) 1.0, (**c**) 1.5 and (**d**) 2.0 UC in [010] or *b*-direction. The blue plane represents the (010) diffraction plane. (**e**) Schematic description of the method of rel-rod mapping by tilting the Ewald sphere in positive and negative directions (+*θ* and –*θ*).

To determine the thickness of a 2D zeolite, we took advantage of the fact that the reciprocal lattice of the nanosheet, which is uniquely defined by the sample thickness, can be easily mapped by electron diffraction. As the thickness of the nanosheets (that is, the number of UCs spanning top and bottom surfaces) decreases, the reciprocal lattice points elongate in reciprocal space to form rod-like structures, which are often referred to as ‘rel-rods'^30^. Figure 2a–d shows reciprocal lattices modelled for MFI nanosheets with thicknesses 0.5, 1.0, 1.5 and 2.0 UC, respectively, (the details of calculations are explained in the Methods section). Since AFM data show that these nanosheets are ∼3 nm thick^5^, we limited our analysis to a maximum thickness of 3.98 nm or 2.0 UC. As can be seen, the rel-rods are sensitive even to fractional UC increments of thickness.

Using the kinematical theory of electron diffraction^31^, the patterns recorded in the TEM can be described as the intersections of the Ewald sphere with the reciprocal lattice (Fig. 2e). The rel-rods of nanosheets can then be discretely mapped by tilting the sheets and acquiring diffraction patterns at each tilt angle (*θ*). Here, to simplify the analysis, the initial orientation (*θ*=0°) of the nanosheets was selected to be along the [010] zone axis, which is normal to the nanosheet surface. A tilt-series of diffraction patterns from a MFI nanosheet was acquired from *θ*= −60° to *θ*=60° with tilt-axis perpendicular to the [010] direction. While any tilt-axis perpendicular to [010] allows rel-rod mapping, knowledge of the axis is critical for accurate analysis. In this experiment, it was determined to be 
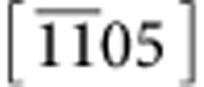
 (see tilt-axis determination in the Methods section and Supplementary Fig. 2).

**Figure 3:**
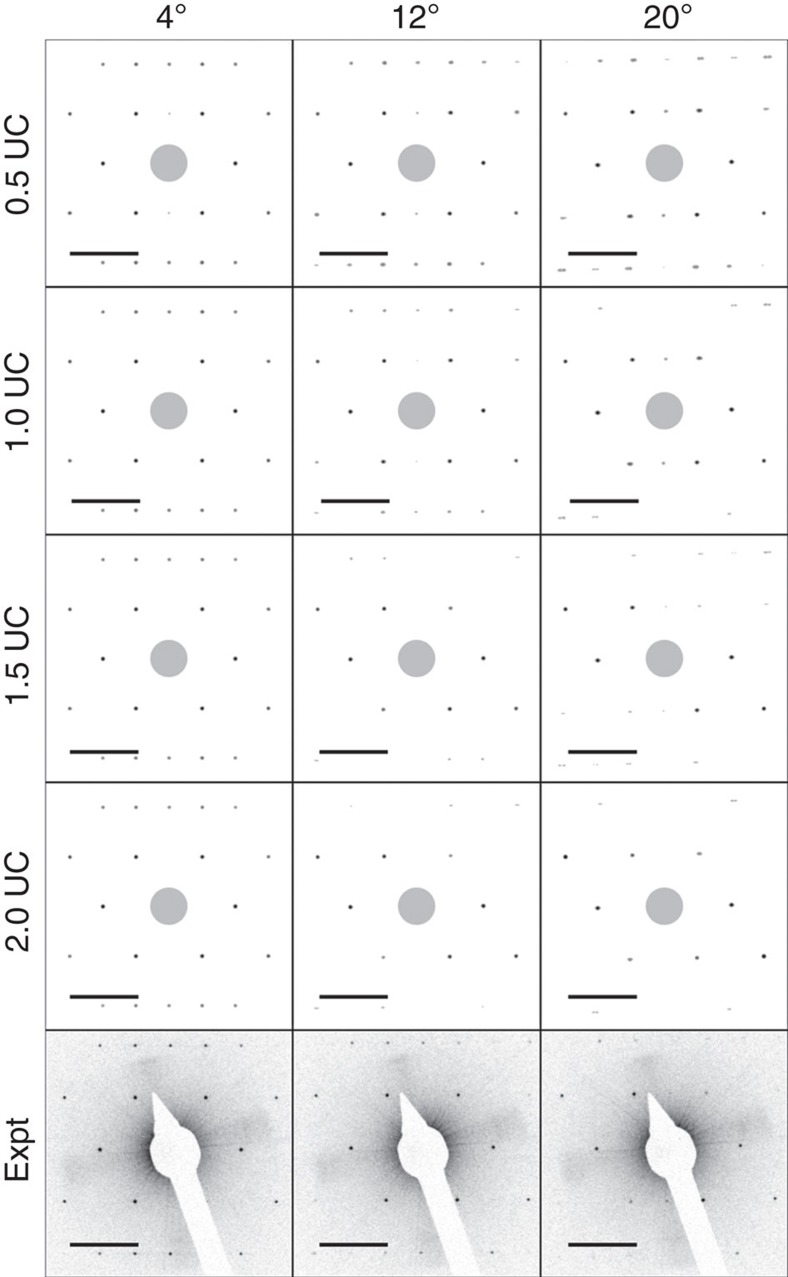
Comparison of experimental and simulated diffraction pattern tilt-series. Simulated diffraction pattern tilt-series (with tilt-axis: 
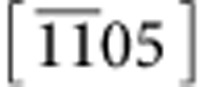
) for MFI zeolite nanosheets of thickness 0.5, 1.0, 1.5 and 2.0 UC. Corresponding experimental (Expt) diffraction patterns of MFI nanosheets are presented in the bottom row. All results are presented in reverse grey-scale colour map for better visibility. Scale bars, 1 nm^−1^. All data sets show clear changes in diffraction pattern contrast as a function of sample tilt.

To correlate the shape of the experimentally mapped rel-rod to the thickness of the nanosheet, we simulated diffraction pattern tilt-series and mapped rel-rods of nanosheets with four different thicknesses: 0.5, 1.0, 1.5 and 2.0 UC. Simulations were performed using the multislice method^32^ and the TEMSIM simulation package developed by Kirkland^21^, using parameters closely matching the experimental conditions (see ‘Multislice simulations' in the Methods section for details). The experimentally obtained tilt-series of diffraction patterns from a MFI nanosheet, along with a set of simulated patterns, are presented in Fig. 3 (additional data are provided in Supplementary Fig. 3).

**Figure 4:**
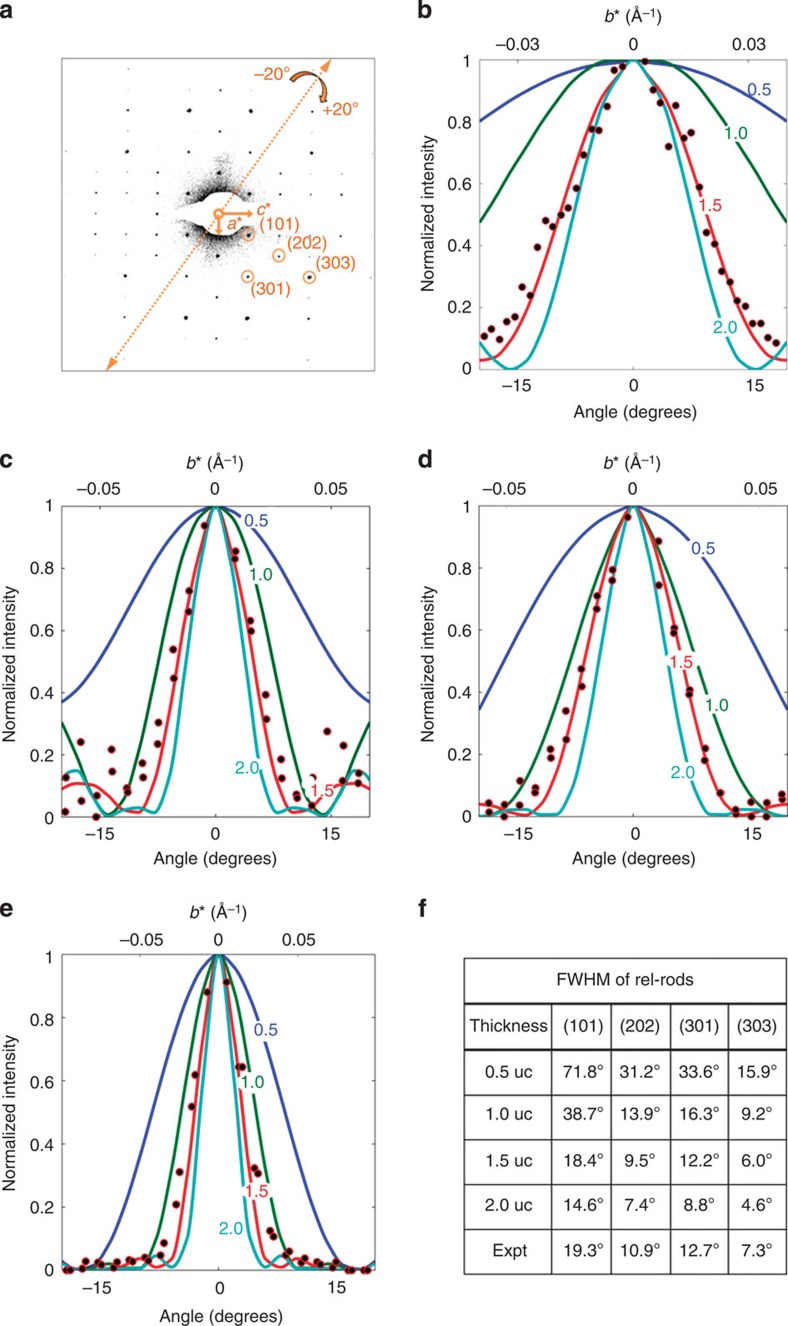
Diffraction spot intensity modulation with tilt. (**a**) TEM electron diffraction pattern taken along [010] zone axis. It is presented in reverse grey-scale colour map for better visibility. The 
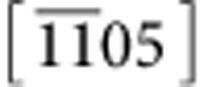
 tilt-axis is indicated with an orange arrow. Diffraction spots used for analysis are circled. The variation in intensity with tilt angle (rel-rod map) of (**b**) (101), (**c**) (202), (**d**) (301) and (**e**) (303) spots are plotted for simulated (lines) and experimental (circles) data, respectively. (**f**) Calculated FWHMs of simulated and experimentally mapped rel-rods show best agreement for a 1.5-UC-thick nanosheet. FWHMs were calculated by fitting a 1D Gaussian function.

To quantify diffraction intensities, the intensity (*I*_*θ*_) of any diffraction spot in the tilt-series was calculated as the volume under a 2D Gaussian function fitted to that spot (see ‘Gaussian fitting' in the Methods section and Supplementary Fig. 4). The same procedure was applied to the simulated tilt-series as well. Moreover, for quantitative analysis of the experimental data, the intensities obtained were corrected for beam damage (see ‘Compensation for beam damage' in the Methods section and Supplementary Fig. 5). For direct comparison of experimentally obtained and simulated rel-rods, they are both normalized to 1 at *θ*=0°. Figure 4b–e shows the resulting experimental and simulated rel-rod maps corresponding to (101), (202), (301) and (303) diffraction spots for the MFI nanosheet. Comparison of full-width at half-maxima (FWHM) of experimentally mapped rel-rods (see Supplementary Fig. 6 for evaluation of FWHM of rel-rods) with the simulated ones is given in Fig. 4f. All the experimental data agree with 1.5-UC-thick nanosheet simulations, regardless of the diffraction spot chosen for analysis. This finding confirms the tentative conclusion made earlier by inspecting X-ray diffraction (XRD) data, comparing them with simulations and combining them with AFM data^5^. In future studies, it would be interesting to compare the single particle data obtained here, with quantitative analysis of bulk XRD measurements (for example, powder diffraction and/or grazing incidence on monolayers).

### Quantification of wrinkling

In addition to observing intensity modulations of diffraction spots with tilt, we also tracked changes in the shapes of the spots, as they are a measure of wrinkling of the MFI nanosheet. The wrinkling of nanosheets in real space corresponds to precession of the rel-rods into ‘cones' in reciprocal space; therefore, the diffraction spot shape changes are particularly pronounced at larger tilt angles (for more details, see Supplementary Fig. 7). It should be noted here that to ensure accurate analysis, it is essential to exclude the possibilities of shape changes due to uncompensated astigmatism of the lenses or slight convergence of the electron beam. Our TEM alignments confirmed that the probe had minimal astigmatism during acquisition. Moreover, we found from simulations (shown in Supplementary Fig. 8) that changes in convergence angle of the electron beam cause changes in the size of diffraction spots at all tilt angles but do not lead to detectable shape asymmetry. Therefore, the changes in diffraction spots' shape observed here can be confidently attributed to wrinkling of nanosheets.

Wrinkling was also detected by HR-TEM images of the MFI nanosheets, obtained along the [010] zone axis, showing that the nanosheet structure is not uniformly in focus. It exhibits in- and out-of focus domains with an average characteristic dimension of *l*_w_ ≈ 20 nm (an example of one such HR-TEM image is shown in Supplementary Fig. 9a).

In order to quantify the wrinkles in the MFI nanosheet, the FWHM of the (011) diffraction spot was measured in reciprocal a*- (FWHM_a*_) and c*- (FWHM_c*_) directions. This spot, which is favourably close to the tilt-axis, is quantifiable within the tilt angle range 18°–50°, even though it was not observed in the diffraction pattern recorded at [010] zone axis with no tilt (*θ*=0°). It should be noted here that selecting the diffraction spot in which to analyse broadening of the corresponding rel-rod is critical, as some of the spots will show splitting instead of broadening with tilt. While the method is still valid, more rigorous analysis of modified rel-rods will be required to determine the level of wrinkling from those diffracted spots.

**Figure 5:**
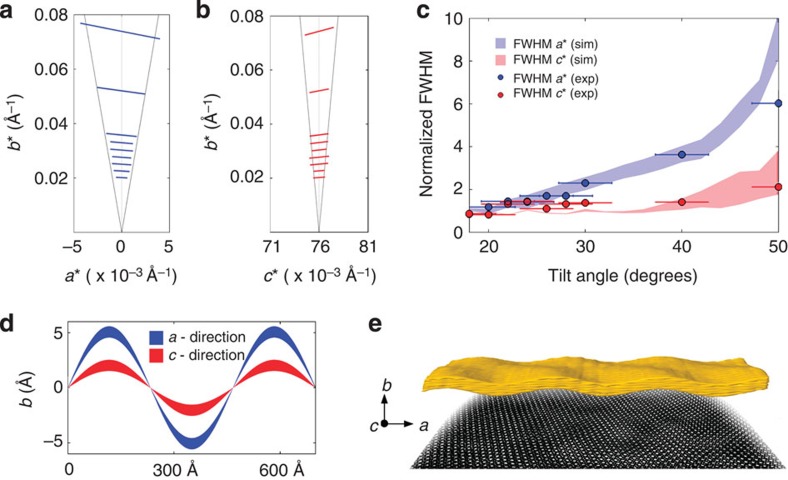
Quantification of diffraction spot shape change with tilt (**a**) a*b* and (**b**) b*c* projections of (011) rel-rods from experimental diffraction patterns of MFI nanosheet. Ewald sphere intersections with rel-rods are plotted as solid blue and red lines. The cones determine the broadening of the rel-rods due to nanosheet wrinkling. (**c**) Comparison of experimental and simulated FWHM_a*_ and FWHM_c*_ of (011) diffraction spot for a 1.5-UC-thick MFI nanosheet as a function of tilt (normalization of FWHM is done with FWHM at *θ*=18°). The error bars represent mechanical accuracy of TEM and holder in tilt angle determination (see ‘Error analysis' in Methods section). (**d**) Two sine waves in *a*- and *c*-directions, superposition of which provides the best description of the wrinkles in these MFI nanosheets. (**e**) Bragg-filtered HR-TEM image of a MFI nanosheet showing variations in intensity across the sheet overlaid with the estimated wrinkled nanosheet model.

The values of FWHM_a*_ and FWHM_c*_ were determined by fitting a 2D Gaussian function to the (011) diffraction spot in each diffraction pattern of the tilt-series as described previously. Broadened due to wrinkling, the (011) rel-rod is then reconstructed in reciprocal space by calculating the line segments in a*- and c*-directions corresponding to intersection of the Ewald sphere and the rel-rod (details are provided in Supplementary Fig. 9b,c). The results are presented in Fig. 5a,b. Projections of this reconstructed rel-rod show that the rel-rod is broadened more in the a*-direction than in the c*-direction, with maximum tilt angles of *θ*_a*_=2.66° and *θ*_c*_=1.18°, correspondingly. This difference in broadening of rel-rod in a*- versus c*-directions indicates that MFI nanosheets wrinkle differently in *a*- and *c*-directions. The greater resistance of the MFI nanosheets to bending in the *c*-direction likely results from the stiff pentasil periodic bond chains that extend along the *c*-direction.

To accurately translate the measured broadening of rel-rods into a measure of MFI nanosheet wrinkling, measured FWHMs of (011) rel-rods are compared with those generated theoretically using multislice simulations. The experimentally determined values of *θ*_a*_=2.66°, *θ*_c*_=1.18° and domain size *l*_w_=20 nm were used to set up an initial model of a wrinkled MFI nanosheet. The structural model is created by shifting all atoms of an ideally flat nanosheet in the *b*-direction (*y*_*i*_) using the superposition of two perpendicular sine waves with a wavelength 2*l*_w_. The new position of atoms in the wrinkled nanosheet can be expressed as:


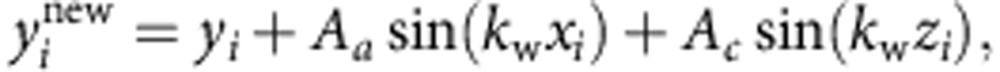


where (*x*_*i*_*, y*_*i*_*, z*_*i*_) are the coordinates of atom *i*, *A*_*a*_=(*l*_w_*/*2)tan(*θ*_a*_) and *A*_*c*_=(*l*_w_*/*2)tan(*θ*_c*_) are amplitudes of sine waves in *a*- and *c*- direction, respectively, and, *k*_w_=*π/l*_w_ (see Supplementary Fig. 9d for details of creating wrinkled nanosheet models). The values of *A*_*a*_ and *A*_*c*_ are then determined from fitting of FWHM_a*_ and FWHM_c*_ of (011) spot in simulated diffraction pattern tilt-series to the corresponding experimental data as shown in Fig. 5c. The best fit resulted in the following values: *A*_*a*_=5.0±0.5 Å, *A*_*c*_=2.0±0.5 Å and *k*_w_=0.013 Å^−1^ (Fig. 5d). The total deviation from flatness for this wrinkled nanosheet is estimated to be in the range of −8 Å to +8 Å (up to 0.04 Å shift in *b*-direction for every 1.0 Å traversed laterally) with wrinkling along the *a*-direction being more pronounced (Fig. 5e). To our knowledge, this is the first time that wrinkling of highly crystalline zeolite nanosheets is quantified.

With MFI nanosheets as an example, we have shown that by using TEM and quantitatively mapping the reciprocal lattice, it is possible to fully characterize the atomic structure of a 2D zeolite, including determination of crystal structure, sheet thickness and the level of wrinkling. We showed that the method is sufficiently simple and robust to be applicable for 2D zeolites, which are fractional multiples of UCs in thickness. This analytical technique of mapping the reciprocal lattice is based on monitoring the modulations in intensity and changes in shape of diffraction spots with tilt, allowing the thickness and wrinkling of 2D zeolites to be determined from a tilt-series of diffraction patterns. This technique does not even require the use of double tilt TEM holders. The issue of beam damage that typically limits any TEM study of zeolites is accounted for and quantitatively incorporated into this method. The method should be applicable to zeolites including aluminosilicates and to other porous materials including metal–organic frameworks. Application of this method on a 2D zeolite with the MFI structure reveals that the exfoliated MFI nanosheets are 1.5 UCs or 3 nm thick, and while they have the same nominal crystal structure as bulk MFI, they are notably wrinkled. The wrinkling is non-uniform, with up to 0.8 nm deviations from flatness. It is possible that the anisotropic flexibility of 2D MFI zeolite nanosheets will have considerable effects on their application as adsorbents, membranes and catalysts, and should be taken into account in future studies.

## Methods

### Materials

MFI nanosheet suspensions in octanol were prepared from multilamellar MFI as reported by Choi *et al*.^8^ followed by exfoliation by melt blending and purification by density gradient centrifugation as described by Varoon *et al*.^5^,^6^. TEM samples were prepared by drop-casting an octanol suspension onto standard 400-mesh TEM copper grids coated with holey carbon film supported on an ultrathin carbon layer (from Ted Pella Inc.).

### Instrumentation

FEI Tecnai G2 F30 (S)TEM equipped with TWIN pole piece (*C*s=2 mm) and a Schottky field emission electron gun operating at 300 kV with extraction voltage of 4,000 V was used for conventional bright-field TEM imaging. Low-dose setup of the microscope was used to minimize beam exposure of the sample between tilts. The emission current during the experiment was 90 μA. Selected area electron diffraction patterns were acquired with an integration time of 8 s at a camera length of 3.7 m on a Gatan 4k × 4k Ultrascan CCD at a 4 × 4 binning to yield a final 1k × 1k pixel^2^ image. HAADF-STEM imaging was performed on FEI Tecnai G2 F30 (S)TEM equipped with S-TWIN pole piece (*C*s=1.3 mm) and a Schottky field emission electron gun operating at 300 kV. HAADF detector collection inner and outer angles were 50 and 200 mrad, respectively.

### Reciprocal lattice modelling

A 3D reciprocal lattice of MFI nanosheet was constructed by plotting the square of the structure factor *F*(**q**), which is defined as 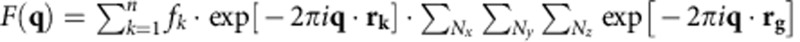, where **q** is a lattice vector in reciprocal space, *f*_*k*_ is the atomic scattering factor, and **r**_**k**_ is the position vector for atom *k* in the UC. The lattice amplitude, which can be simplified to 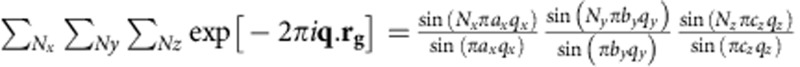, depends on the number of UCs (*N*_*x*_, *N*_*y*_ and *N*_*z*_) in the crystal in the *x-, y*- and *z*-direction, respectively, and on real space position vector **r**_**g**_ of each UC. The orthorhombic UC of MFI (*a*=20.09 Å, *b*=19.74 Å, *c*=13.14 Å, *α*=*β*=*γ*=90°) was used to create models of MFI nanosheets (*N*_*x*_=10 UC; *N*_*y*_=0.5, 1.0, 1.5 and 2.0 UC and *N*_*z*_=15 UC) and the respective reciprocal lattices.

### Tilt-axis determination

Diffraction spots move upon tilting. We track the motion paths of higher-order diffraction spots moving parallel to each other throughout the tilt (Supplementary Fig. 2a,b). Crystallographically equivalent diffraction spots on either side of the tilt-axis move in opposite directions. The common direction in the diffraction pattern that satisfies symmetric movements of equivalent diffraction spots is therefore the tilt-axis. The estimated tilt-axis for the experimental data analysed here is the 
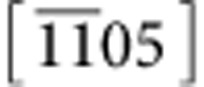
 crystallographic direction, which passes through the plane of the nanosheet.

### Multislice simulations

Supercells of MFI nanosheets with dimensions 1,100 Å × 1,100 Å in *a*- and *c*-directions, respectively, and thickness of 9.94 Å (0.5 UC), 19.89 Å (1.0 UC), 29.83 Å (1.5 UC) and 39.78 Å (2.0 UC) in the *b*-direction were created from an orthorhombic MFI UC. Nanosheet models were tilted using rotation matrices from *θ*=−60° to 60° in steps of 2° to generate the atomic positions for tilted nanosheet structures. Diffraction pattern tilt-series were simulated from these models using the TEMSIM multislice simulation package. A 4 k × 4 k pixel^2^ grid was used in these simulations, which provide pixel sizes of 0.27 Å per pixel in *a*- and *c*-direction. The electron probe was generated using a spherical aberration coefficient *C*_s_=2 mm, convergence angle *α*=0.02 mrad, defocus Δ*f*=800 Å and beam energy *E*_0_=300 keV.

### Gaussian fitting

32 × 32 Pixel sections from the peaks of interest (Fig. 4a) were extracted from the experimental and simulated diffraction patterns using a custom script in Matlab. Using the curve fitting algorithm in Matlab, a 2D Gaussian function, defined as 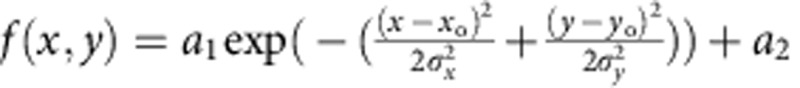 was fitted to the entire 32 × 32 pixel region, using *σ*_*x*_, *σ*_*y*_, *a*_1_*, a*_2_*, x*_0_ and *y*_0_ as fitting parameters (shown in Supplementary Fig. 4). This fitting also estimates the background level in diffraction patterns due to the central beam with the parameter *a*_2_. Intensity of diffraction spot at each tilt angle is the integrated intensity of the fitted 2D Gaussian function (*I*_*θ*_=2*πa*_1_*σ*_*x*_*σ*_*y*_). The broadening of the diffraction spot is measured in two directions as: 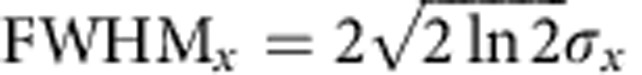 and 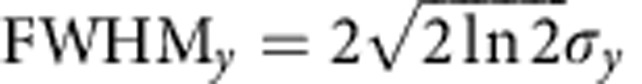.

### Compensation for beam damage

MFI nanosheets, such as all zeolites, are electron beam sensitive. Therefore, diffraction patterns were acquired under a low-dose condition. Loss of diffraction intensity was compensated by separately measuring the reduction of diffraction spot intensities at *θ*=0° as a function of dose within the first 100 min of exposure (typical tilt experiments take ∼100 min). As shown in Supplementary Fig. 5, in experiments with emission current of ∼90 μA, loss of diffraction intensity with time due to amorphization was found to be linear with slopes: *k*_{101}_=2.8 × 10^−5^ min^−1^, *k*_{202}_=8.7 × 10^−4^ min^−1^, *k*_{301}_=7.2 × 10^−4^ min^−1^ and *k*_{303}_=4.0 × 10^−3^ min^−1^. In order to compensate for the effects of amorphization, the diffraction spot intensities obtained in each tilt experiment are adjusted to damage-free intensity by multiplying them by a factor of (1−*kt*)^−1^, where *t* is the corresponding time at which they were acquired.

### Error analysis

(101) and 
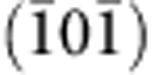
 being crystallographically equivalent spots are expected to have same intensity values at all time points for tilt angle *θ*=0°. However, a mismatch in experimental intensity values was observed, which is attributed to the limited mechanical precision of the TEM goniometer in tilting the nanosheets to a specific tilt angle (*θ*). The relative error in intensity measurement i.e., 
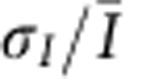
, was calculated from the recorded measurements to be 0.1, where 
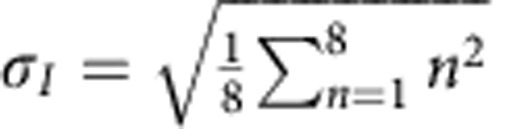
, 
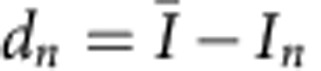
 and 
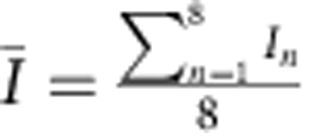
. The damage-free intensities of (101) and 
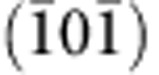
 spots from experimental diffraction patterns are listed in Supplementary Table 1. To correlate the error in intensity to error in *θ*, the expected values for intensity modulation of crystallographically equivalent (101) and 
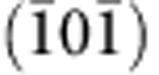
 for a 1.5-UC-thick nanosheet (Supplementary Fig. 10a) was determined. This known intensity modulation was then used to estimate the maximum possible error in tilt angle (|Δ*θ*|) to be 3.4° (Supplementary Fig. 10b), as indicated in Fig. 5c with the horizontal error bars.

## Author contributions

P.K. conceived, executed and analysed TEM experiments. K.V.A. synthesized the zeolite samples. P.K., M.T. and K.A.M. wrote the manuscript. M.T. and K.A.M. conceived and directed the project. All the authors participated in the discussion and interpretation of data, read the manuscript and provided input.

## Additional information

**How to cite this article:** Kumar, P. *et al*. Quantification of thickness and wrinkling of exfoliated two-dimensional zeolite nanosheets. *Nat. Commun*. 6:7128 doi: 10.1038/ncomms8128 (2015).

## Supplementary Material

Supplementary Information Supplementary Figures 1-10 and Supplementary Table 1
