# Supplementary material for: Quantification of thickness and wrinkling of exfoliated two-dimensional zeolite nanosheets
Source: Nat Commun. 2015 May 11;6:7128. doi: 10.1038/ncomms8128 (PMC4432588; doi:10.1038/ncomms8128)
Supplement: Supplementary Information — Supplementary Figures 1-10 and Supplementary Table 1 [file ncomms8128-s1.pdf]

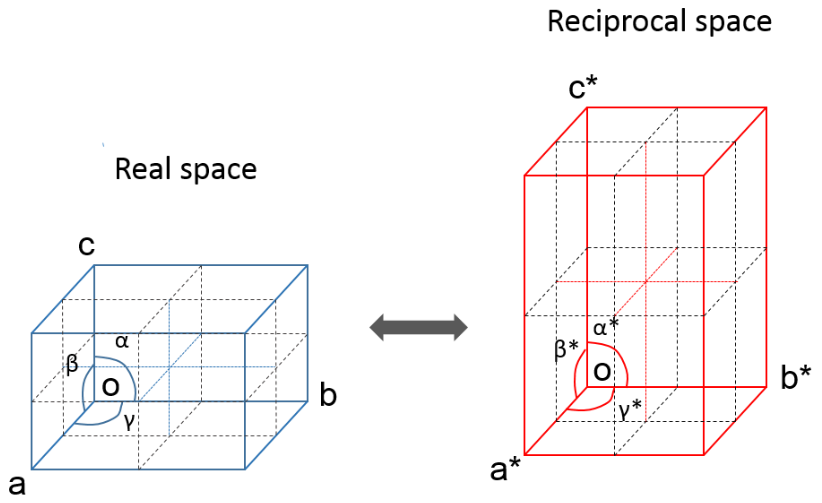

Supplementary Figure 1. Orthorhombic unit cell of MFI (a=20.09 Å, b=19.74 Å, c=13.14 Å, α=β=γ=90°) and its corresponding reciprocal lattice (a*=0.049 Å^{-1}, b*=0.051 Å^{-1}, c*=0.076 Å^{-1}, α*=β*=γ*=90°). Three mutually perpendicular mirror planes marked by dashed lines.

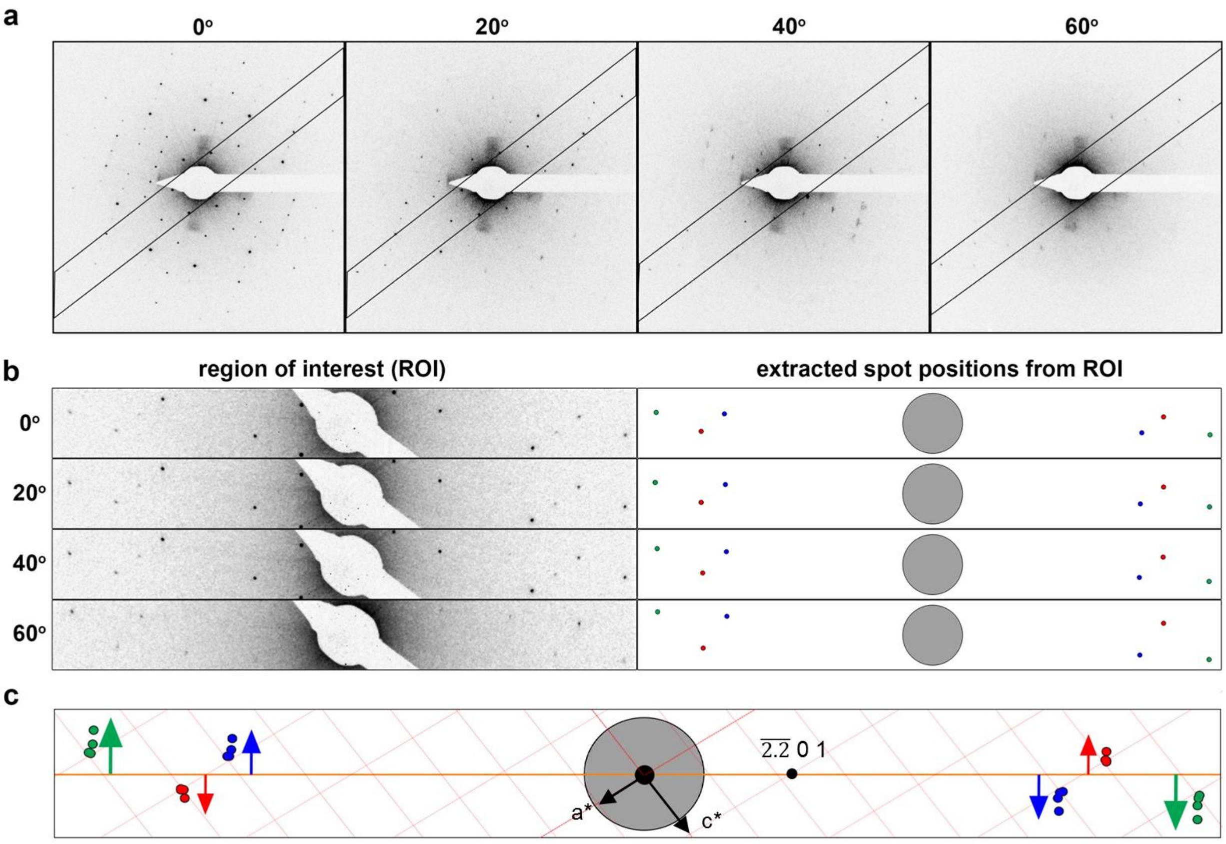

Supplementary Figure 2. (a) Tilt-series of diffraction patterns acquired at camera length Lc = 1.9 m to image higher order diffraction spots. Diffraction spots in close proximity of the tilt-axis are visible across the complete tilt range of 0°-60°. This allows us to estimate the tilt-axis. (b) Region of interest (ROI) marked in (a). Positions of six higher order diffraction spots used in analysis are marked (green, red and blue) on the right-hand panel. (c) ROIs from 0°-60° are superposed to track the motion paths of all six diffraction spots selected in (b). Crystallographically equivalent spots have the same color, and arrows point in the direction of movement of spots with tilt. The tilt-axis is the common direction in reciprocal lattice (in a*c*-plane) that is perpendicular to motion paths of all spots. It is calculated to be [$\overline{11}$ 0 5].

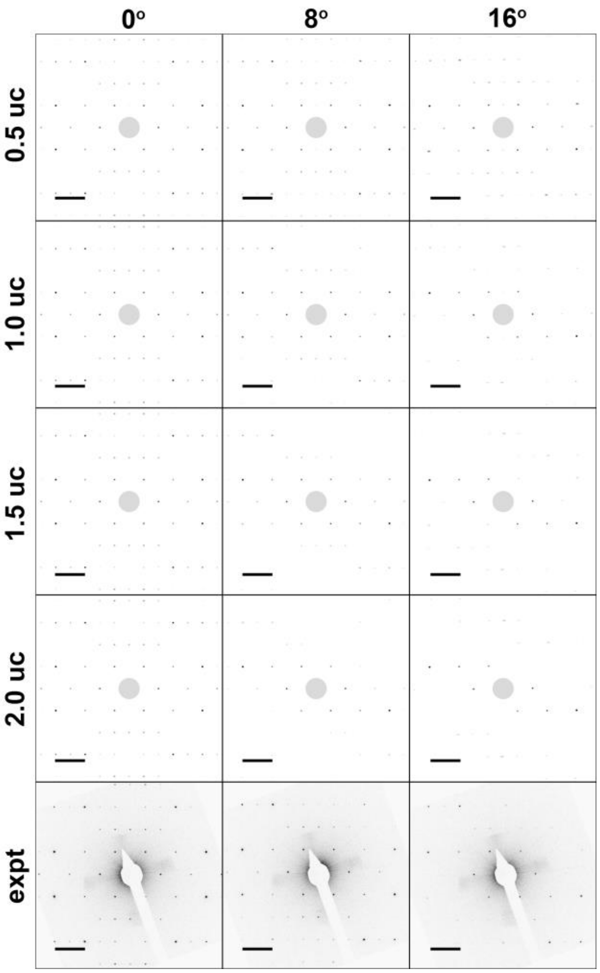

Supplementary Figure 3. Additional diffraction patterns at steps of 0°, 8°, and 16° from the same tilt-series (tilt-axis: [$\overline{11}$ 0 5]) of diffraction patterns presented in Fig. 3 of the main text. Scale bar: 1 nm^{-1}.

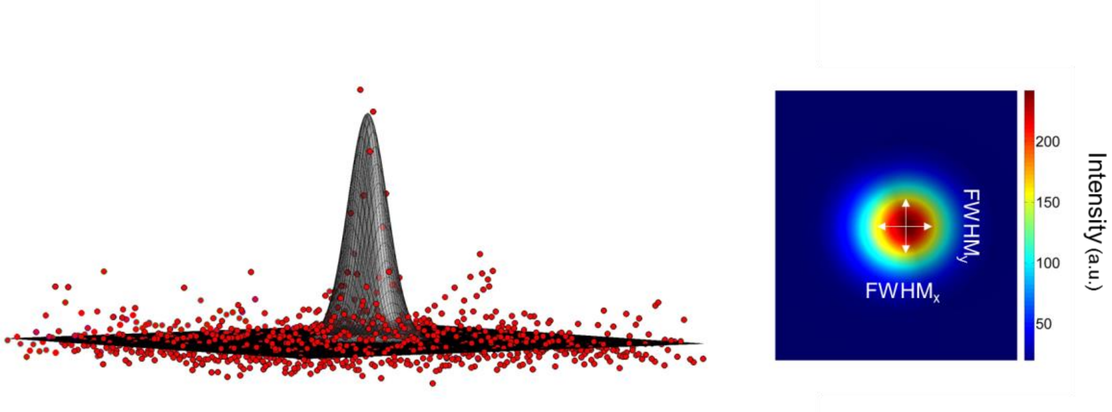

Supplementary Figure 4. An example of a 2D Gaussian function used to fit experimental data (in red) for calculating the diffraction spot intensity (volume under the Gaussian surface) and for determining the FWHMs of the spot.

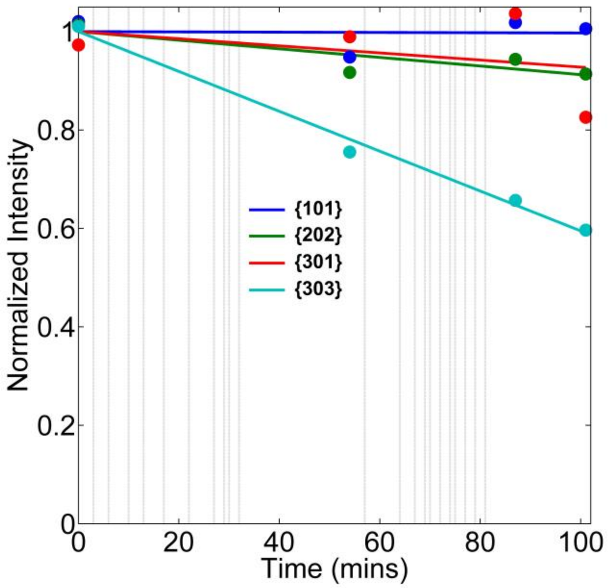

Supplementary Figure 5. Diffraction spot intensity decay as function of beam exposure time. Due to non-uniform electron beam damage {101}, {202}, {301} and {303} spot intensities decay with different rates. Vertical lines correspond to the time points at which the tilt-series diffraction patterns were acquired.

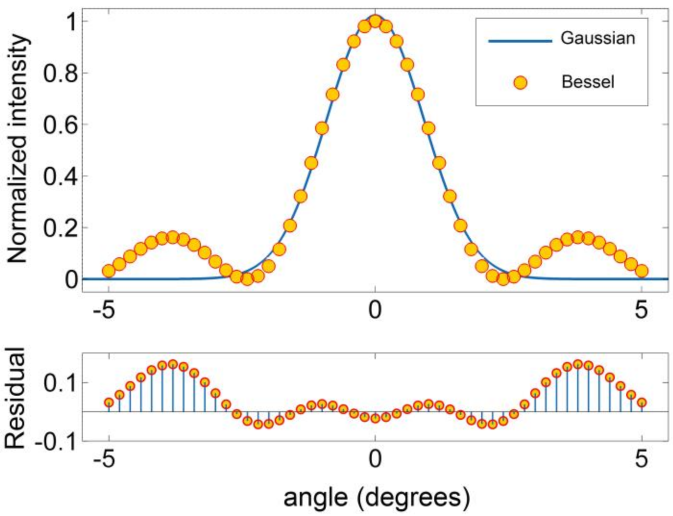

Supplementary Figure 6. Rel-rods of thin crystalline specimens can be described using Bessel functions of first kind. The FWHM of the primary peak of the Bessel function can be estimated by a 1D Gaussian function fit. Residual intensity (or difference) plot shows that the primary peak of the Bessel function is accurately estimated by a simple Gaussian function.

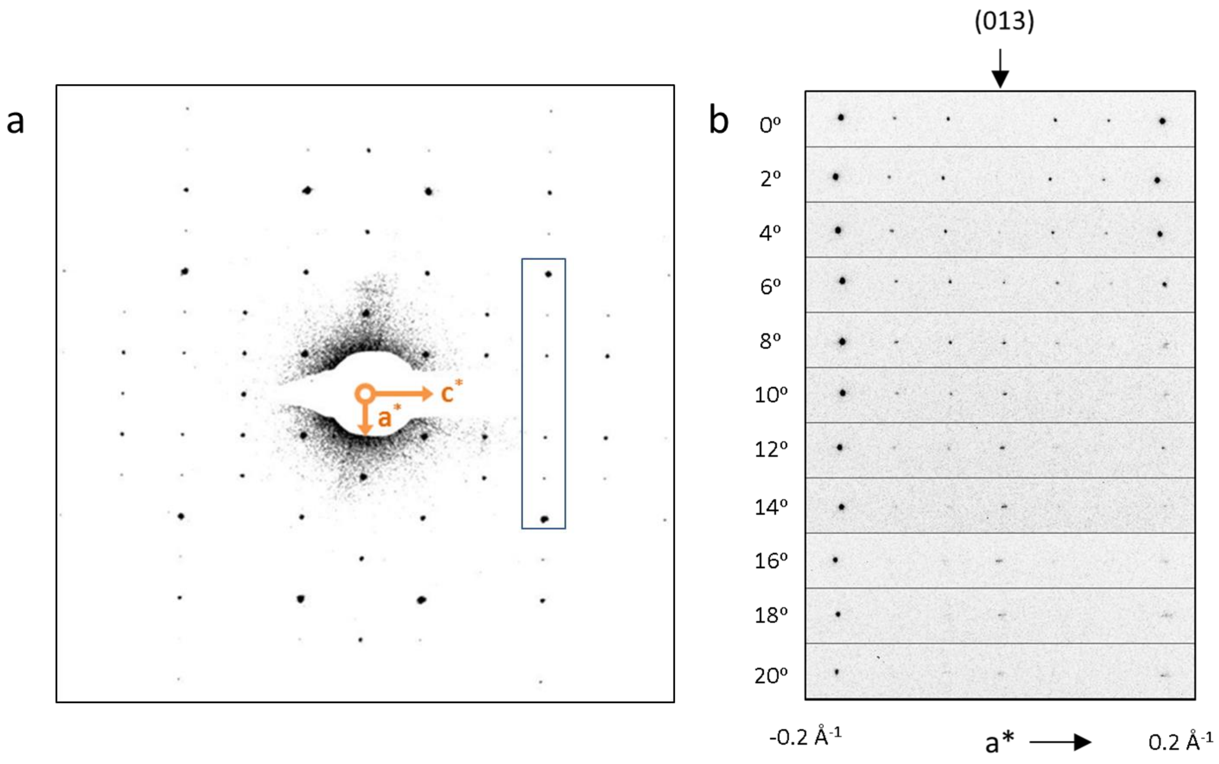

Supplementary Figure 7. (a) Diffraction pattern from MFI nanosheet precisely aligned to the [010] zone axis. (b) Sections of experimental diffraction pattern tilt-series as marked in (a) show variations in peak intensity and changes in shape of the (013) diffraction spot.

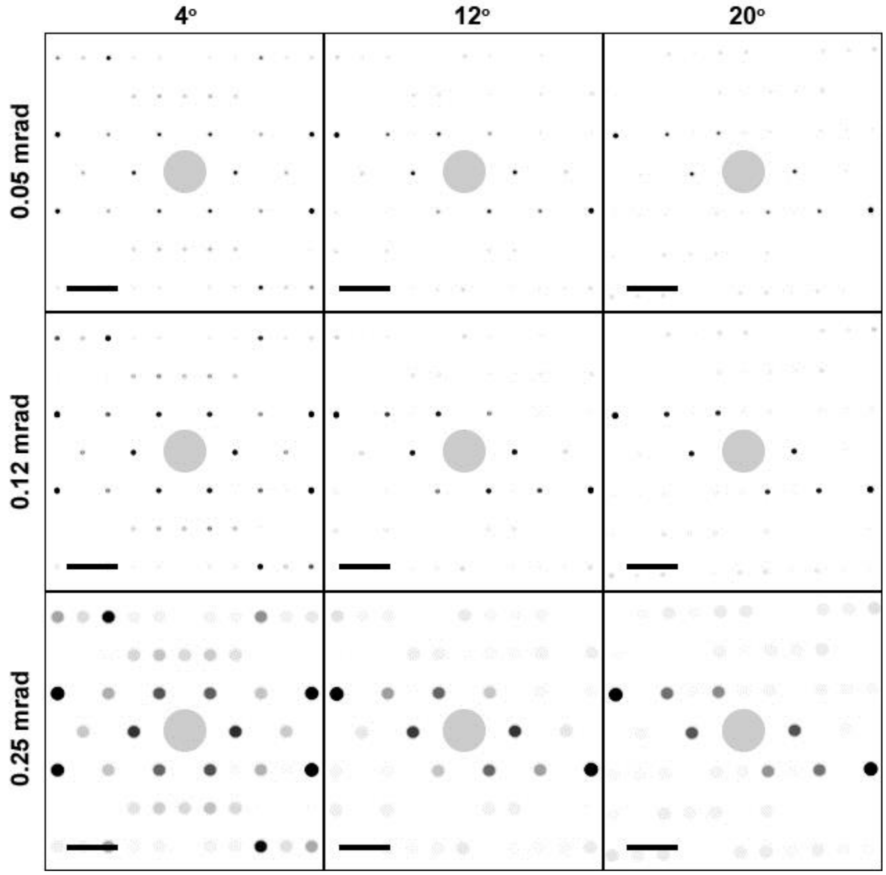

Supplementary Figure 8. Multislice simulated diffraction pattern tilt-series of 1.5 uc thick MFI nanosheet for three different electron probe convergence angles: 0.05, 0.12 and 0.25 mrad. Increasing convergence angle causes the spot width to increase while maintaining its circular shape. No asymmetric changes in spot shape are seen upon increasing the convergence angle. Scale bar is 1 nm^{-1}.

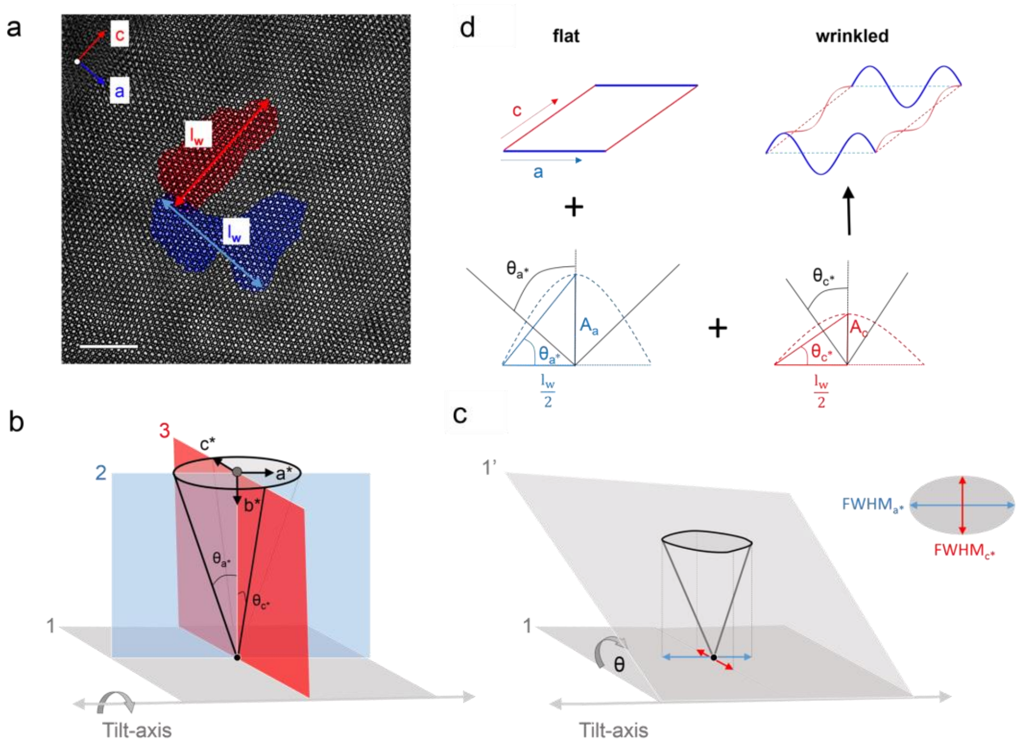

Supplementary Figure 9. Broadening of rel-rods due to wrinkling can be described using rel-rods of non-wrinkled MFI nanosheets with additional tilts of 0-θ_{a*} in a*- and 0-θ_{c*} in c*-direction, which results in rel-rods with elliptical cone shape. (a) Bragg filtered HR-TEM image of MFI nanosheet showing in- and out-of focus domains of about 20 nm in size (in both a- and c-direction). Scale bar here is 10 nm. (b) Schematic description of intersection of three planes with (011) rel-rod cone: Ewald sphere (gray plane, labeled 1), a*b* plane (blue plane, labeled 2), b*c* plane (red plane, labeled 3). (c) Schematic description of intersection of Ewald sphere with broadened rel-rod at tilt angle θ. (d) A model of a wrinkled MFI nanosheet generated from a flat sheet by superposing two perpendicular sine waves with wavelength of $2l_w$ = 40 nm and amplitudes: $A_a = (l_w/2)\tan(\theta_{a*})$ in a-direction, $A_c = (l_w/2)\tan(\theta_{c*})$ in c-direction. The rel-rod tilt broadening due to wrinkling is shown by solid black lines in each direction and can be described by angles: θ_{a*}, θ_{c*}.

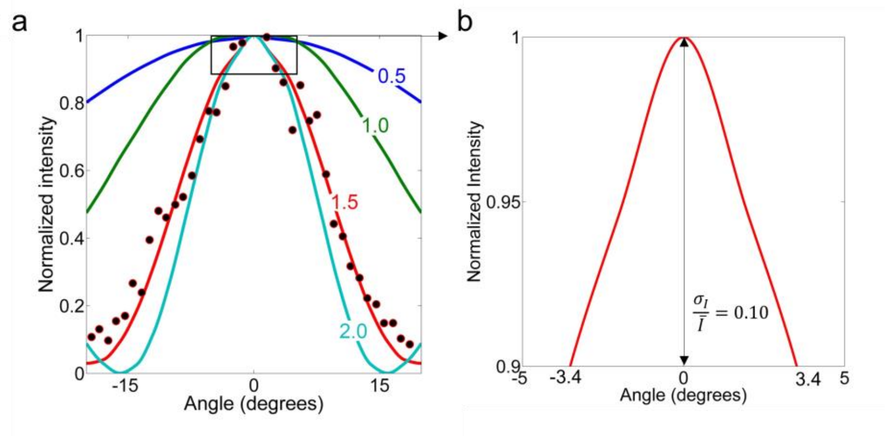

Supplementary Figure 10. (a) Intensity modulation of (101) diffraction spot as shown for simulated nanosheets (0.5, 1.0, 1.5 and 2.0 uc thickness) and experimental data (black scatter data) (b) Intensity modulations for (101) diffraction spot for 1.5 uc thick nanosheet from the boxed region in (a) showing the relation between relative intensity error (0.10) and tilt angle.

Supplementary Table 1 : Damage free Intensity (x 10^4) at θ = 0°		
Time (t)	(101)	$(\bar{1}0\bar{1})$
0	I_1=7.7	I_2=6.2
57	I_3=6.7	I_4=6.3
84	I_5=7.2	I_6=6.8
101	I_7=7.9	I_8=5.9
